# ‘Microcosm of the Pacific’: Colonial encounters at the Central Medical School in Fiji

**DOI:** 10.1017/mdh.2024.10

**Published:** 2024-04

**Authors:** Hohee Cho

**Affiliations:** Faculty of History, University of Oxford, 47 Banbury Road, Oxford, OX2 6PE, UK

**Keywords:** Central Medical School, Native Medical Practitioner, Medical Education, British Empire, Pacific Islands, Fiji

## Abstract

While larger British colonies in Africa and Asia generally had their own medical services, the British took a different approach in the South Pacific by working with other colonial administrations. Together, colonial administrations of the South Pacific operated a centralised medical service based on the existing system of Native Medical Practitioners in Fiji. The cornerstone of this system was the Central Medical School, established in 1928. Various actors converged on the school despite its apparent isolation from global centres of power. It was run by the colonial government of Fiji, staffed by British-trained tutors, attended by students from twelve colonies, funded and supervised by the Rockefeller Foundation, and jointly managed by the colonial administrations of Britain, Australia, New Zealand, France and the United States. At the time of its establishment, it was seen as an experiment in international cooperation, to the point that the High Commissioner for the Western Pacific called it a ‘microcosm of the Pacific’. Why did the British establish an intercolonial medical school in Oceania, so far from the imperial metropole? How did the medical curriculum at the Central Medical School standardise to meet the imperial norm? And in what ways did colonial encounters occur at the Central Medical School? This article provides answers to these questions by comparing archival documents acquired from five countries. In doing so, this article will pay special attention to the ways in which this medical training institution enabled enduring intercolonial encounters in the Pacific Islands.

## Introduction^1^

Medical education in the South Pacific began as an imperial policy and developed into an unusual intercolonial project that enabled colonial encounters. Fuelled by the desire for a cost-effective method of medical governance, the Central Medical School (CMS) was established in 1928 to provide medical education to Pacific Islanders. It was a result of cooperation between colonial powers that would otherwise be in competing relationships. The CMS was run by the colonial government of Fiji, taught by British tutors, filled with students from across the Pacific, financially supported by the Rockefeller Foundation, and jointly operated by the colonial administrations of Britain, Australia, New Zealand, France, and the United States. This article approaches the establishment of the CMS from an imperial history perspective rather than using a national or colonial framework. The focus of this article is on the period leading up to the establishment of the CMS and its early years between the 1920s and 1930s.

The context of the British Empire is crucial in understanding the intercolonial cooperation in the South Pacific, known as Oceania. It is an excellent frame to study intercolonial relations, as every type of colonial governance of the British Empire was represented during the interwar years. Most prominently, there were self-governing white settler colonies in Australia and New Zealand, in other words, dominions. Apart from these dominions, British colonies were directly managed under the Western Pacific High Commission (WPHC). The High Commissioner for the Western Pacific was, at the same time, the Governor of Fiji, a Crown colony where the WPHC was based. Under the umbrella of the WPHC, the Gilbert and Ellice Islands Colony (today’s Kiribati and Tuvalu, previously the Gilbert and Ellice Islands Protectorate) and the British Solomon Islands Protectorate were governed through the Resident Commissioners. The WPHC also co-administered the Anglo-French condominium of New Hebrides (today’s Vanuatu) with France. Tonga was not a formal colony, maintaining an independent monarchy, but it came under the protection of the British Empire through the Treaty of Friendship. Nauru was a co-mandated territory between Britain, Australia, and New Zealand. It was administered by Australia in practice. In addition, Australia had the territories of Papua and New Guinea. New Zealand controlled Western Samoa, the Cook Islands, Niue, and Tokelau (previously the Union Group). Even Fiji, a Crown colony, had a dependency, Rotuma. Lastly, the British had overseas territories such as the Pitcairn Islands. This article uses the colonial names of each country to reflect the historical units of governance during the period this article examines.

**Figure 1.**
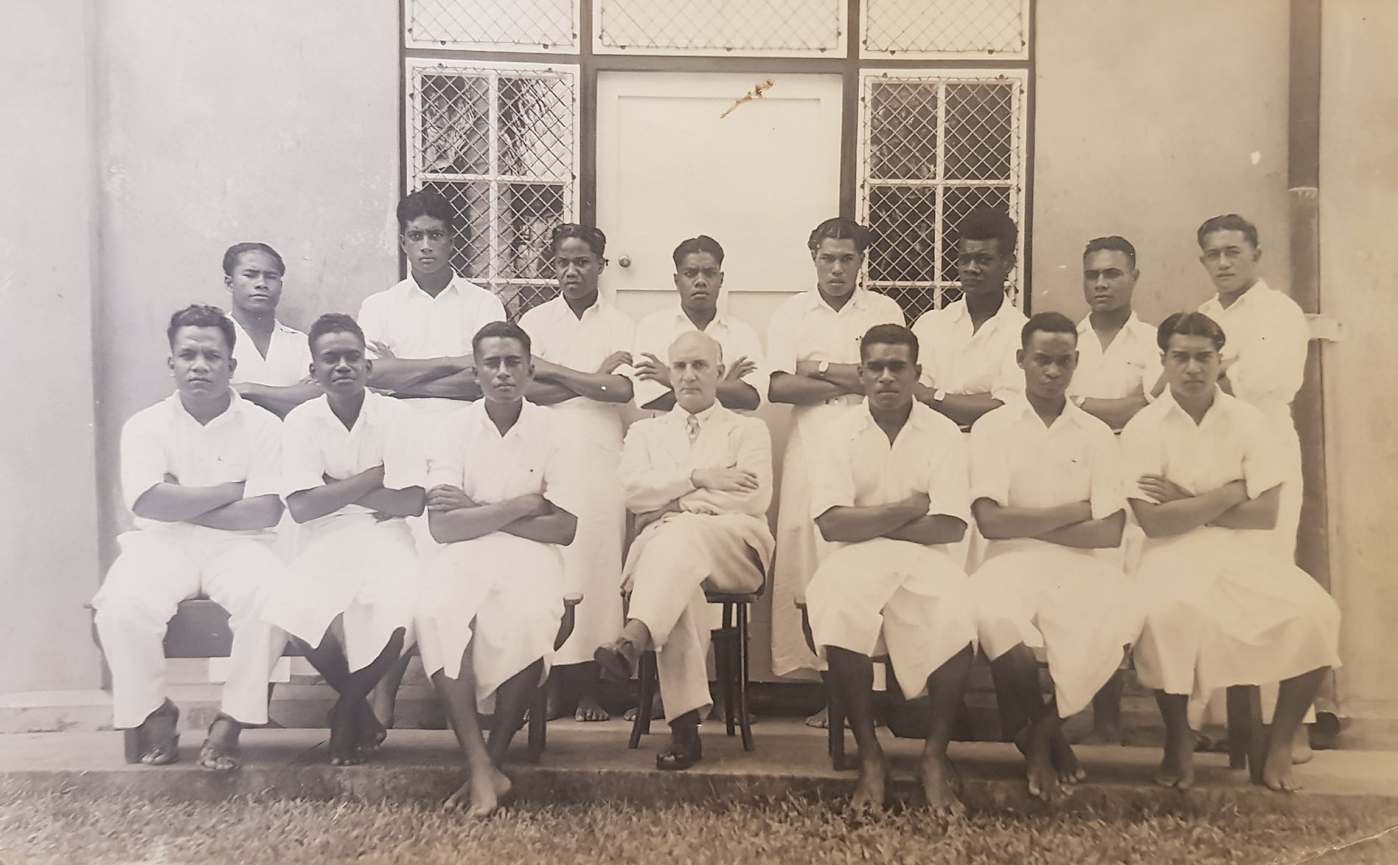
David Hoodless and first-year students from Fiji, Western Samoa, American Samoa, Tonga, Gilbert and Ellice Islands, Solomon Islands, and Cook Islands. First row, second from left: John Wesley Kere. Second row, second from left: Ratu Mara. [Margaret Guthrie’s Private Collection].

Together, the diverse set of administrations in the Pacific established and operated the CMS, where they would train indigenous doctors for their colonies. As Figure 1 illustrates, students from across the Pacific formed a cohort and trained together. The imperial nature of the school reveals that participating administrations shared a common cause for promoting indigenous health, which would ultimately contribute to imperial interests. Although it did not offer a full medical degree, the CMS still developed to meet the imperial norm of medical education. By comparing the cases of each country and the development of medical education over time, this article draws a fuller picture of how such an intercolonial project operated. To analyse the nature of intercolonial cooperation at the CMS from a balanced perspective, this paper brings together archival sources on each participating country from the national archives of the United Kingdom, United States, Australia, New Zealand, and Fiji. It also draws on documents from the Western Pacific Archives and the Rockefeller Archive Center.

Despite the CMS being a fascinating case of an intercolonial institution, the literature on its history has not been very rich. Existing research has briefly noted the intercolonial character of the CMS but has largely treated it as a context for discussing other subjects, such as medical services or the medical profession.^2^ This paper will show that the Pacific Islands connected, cooperated, and competed with each other through the CMS. Further, this paper argues that the CMS was a space for colonial encounters in the South Pacific that allowed imperial authorities to test racial notions on the Pacific Islanders. In doing so, it aims to contribute to the understanding of medical education beyond national boundaries and the scholarship on the British Empire in the Pacific world.

## Establishing the Central Medical School

The genesis of medical education in the Pacific Islands can be traced through its growing links with the British Empire. Records of major epidemics in the Pacific Islands predate the British colonial period. In Fiji, for example, *lila balavu* (wasting sickness) and *cokadra* (dysentery) were reported to be introduced by the first and second European ships arriving in Fiji.^3^ Later, the mobility inherent in being part of the British Empire posed a further threat to indigenous health. The most notable epidemic started with Fiji’s cession to the British Empire in 1874, when the King of Fiji, Ratu Seru Epenisa Cakobau, visited Sydney to finalise the event. Upon return, his delegation brought back measles that ended up killing nearly a quarter of Fijians in 1875. Soon after, intra-imperial migrant labour risked flows of infectious diseases. The first ship carrying indentured labourers from India, *Leonidas*, arrived in 1879 with smallpox and cholera on board. Fijians, who had not yet fully recovered from the devastating measles epidemic, were alarmed by the additional introduction of new diseases.^4^

In a desperate attempt to protect Fijians from potential waves of epidemics, Fiji’s Chief Medical Officer, Dr William MacGregor, requested the establishment of the Suva Medical School (SMS) in 1885. In the meantime, many more ships carrying Indian indentured labourers had arrived in Fiji, which planted fears of imported diseases in the so-called ‘virgin soil’.^5^ Medical education was, therefore, a pragmatic response to the threat of newly introduced diseases. Because the prevention of epidemics was in the colonial government’s interest, all students were government scholars. Their tuition, accommodation, food, and clothing were covered at an annual cost of £25 per student. Admissions were made through an annual entrance examination, from which four students were selected for each cohort.^6^

The primary purpose of establishing the SMS was to train Fijian youths as vaccinators. Within a few years, the Fiji government saw an opportunity to make use of medical students as subordinate staff of the Colonial Medical Service. Graduates of the SMS would then qualify as Native Practitioners, appointed to local districts for the primary care of the indigenous population. In practice, they were called Native Medical Practitioners (NMP). The NMP Ordinance of 1888 defines their duties as including inspection, treatment, vaccination, and writing reports. NMPs were required to visit each town in their district at least once a month and submit daily diaries and quarterly reports detailing their work. Fiji asked them to pay special attention to sanitation and any signs of epidemic outbreaks. If necessary, they were assigned an assistant who would carry their luggage and cultivate food plantations. At this time NMP salaries started at £18 per annum, with an annual increase of £2 to £50 depending on their performance. By comparison, fully qualified European medical officers received a minimum of £350 a year.^7^ Another account reported that NMPs received a minimum of £5 per year as provincial vaccinators.^8^ Exceptionally, one Fijian NMP who served in the Gilbert Islands was so highly appreciated that he received £250 with a living allowance of £50 per annum.^9^

The expansion of the SMS started in the 1920s as a colonial strategy to prevent long-term population decline and promote a healthy labour force. More medical staff were needed in the Pacific Islands after a series of critical epidemics. The most devastating one was the 1918 influenza pandemic. Western Samoa suffered greatly from the influenza pandemic that killed approximately 22% of the entire population.^10^ Fiji lost 5.6% of its population and one-sixth of the indigenous medical staff who took care of the people.^11^ Overall population decline across Oceania was evident. For example, the Fijian population fell from 200,000 in 1870, before it joined the British Empire, to 83,000 in 1919, after the influenza pandemic.^12^ The situation was worse in Hawai‘i. Seth Archer points out that introduced diseases were a major factor in the decline of the Hawaiian population from 500,000 in 1778 to less than 50,000 a hundred years later.^13^ Amid such rapid population decline, the WPHC started to envision a centralised medical governance comprising Fiji, the Solomon Islands, the Gilbert and Ellice Islands, New Hebrides, and Tonga. This was to avoid the worst-case scenario: a ‘race extinction’.^14^

The need for a larger NMP Service was particularly apparent in Fiji, which required a healthy workforce to keep the sugar plantation economy running. Norma McArthur’s study showed that although the 1918 influenza pandemic left a great impact on the Fijian population, the overall population in Fiji started to increase in the 1920s, particularly the Indian population.^15^ This article will use the term ‘Indians’, as used by the CMS authorities at the time, to distinguish this group from indigenous Fijians and to reflect on the historical racial categorisation and colonial racial perceptions.^16^ Many first-generation Indian labourers settled in Fiji even after indenture was abolished in 1917. In 1924, the number of settled Indians in Fiji reached 60,000, matching the size of the estimated number of Fijians. The existing capacity of an average of four students per cohort at the SMS was simply insufficient to cover the entire population.^17^ During the interwar years, the Indian population continued to grow while the Fijian population decreased. In 1934, of the estimated 95,000 individuals in Fiji, 78,000 were ethnically Indian.^18^ These Indian workers were the ones most in need of specialist medical care in Fiji. Without the government’s support, they could not receive sufficient medical attention.^19^ In response to the demand, the WPHC agreed that a larger NMP service was needed. The system could even be exported to other Pacific Islands. The connection to India, another British colony, was not only one of the reasons for the foundation of the SMS but also became a factor in expanding medical education.

Although the British realised the need for more medical staff, they did not want to invest too much of their own capital in indigenous healthcare. Hence, as early as 1922, the WPHC communicated with the Rockefeller Foundation (RF) to provide £10,000 to establish a new and enlarged medical school for British colonies. The new school’s widely imperial character and the possibility of pooling NMPs in the region were put forward as marketing points.^20^ At this stage, the RF was not very responsive. The WPHC also did not make any further effort. This shows that medical education was approached from an imperialist angle, only providing the bare minimum to the colonial population to preserve as many British resources as possible.

A second attempt to gain RF funding was made in 1924 by Western Samoa, a New Zealand territory. Their plea to the RF was that Western Samoa was ‘anxious to keep Samoa for Samoans’ but that it was impossible unless ‘they become a healthy and increasing race’.^21^ It is interesting that the Western Samoan administration, after greatly failing Samoans in the 1918 influenza epidemic, used the expression ‘Samoa for Samoans’, which was a popular Mau slogan, a Samoan independence movement in the late 1920s. The Division of Medical Education of the RF was still sceptical about the project, questioning whether it would be effective given the remoteness and small population of the Pacific Islands. More importantly, the RF preferred to fund university-level medical colleges in more populated countries, such as the recently opened Peking Union Medical College in China. Medical schools for lower qualifications, such as the NMP diploma, were considered not worth investing in.^22^

The project gained momentum in 1925 when the third initiative came from Tonga. It was timely because the Colonial Hospital in Suva was renovated as the Colonial War Memorial Hospital in 1924, becoming the largest medical centre in the Pacific Islands, equipped with 100 beds and modern facilities. Since the SMS was attached to the hospital, this renovation offered ample resources for advanced medical education.^23^ Following a discussion with the British Agent and Consul, the Premier of Tonga proposed WPHC cooperation with other Pacific Islands to establish a joint medical school in February 1925.^24^ The High Commissioner for the Western Pacific welcomed the proposal. He communicated with each respective administration under the WPHC and commenced official talks with New Zealand for their territories.^25^ The last remaining step was to obtain Colonial Office approval for finances in case the RF did not fund the project.^26^ In the meantime, Western Samoa sent two students to the SMS in 1925 to train NMPs as soon as possible.^27^

The RF eventually agreed to a financial contribution after the WPHC (including Tonga) and New Zealand territories had already agreed to establish a larger medical school. A continuous appeal by Dr Sylvester M. Lambert, the RF representative in the South Pacific, helped the decision. Lambert had been conducting medical campaigns in the South Pacific.^28^ His work led him to travel extensively around the Pacific Islands, where he learned that the geographically scattered environment prevented a cohesive operation of medical services. Lambert argued from an imperial angle that ‘it would be Imperially unsound and a short-sighted policy to refrain from establishing a Central Native Medical School even at double the initial cost to that now proposed.’^29^ Because he identified preventable diseases as the main cause of depopulation, training more NMPs and centralised control of the NMPs across Oceania seemed to be the solution to better healthcare.^30^

To persuade the RF to provide funding, Lambert and the WPHC presented three justifications. It was not a simple fact that the indigenous population was decreasing due to introduced diseases. First, there was a sense of moral responsibility for the colonial administrations to care for indigenous welfare. Peoples whom Europeans considered primitive, mostly Melanesians, were victims of large-scale slaughter and forced slavery on plantations, a practice known as blackbirding. The ‘decline of custom’ was a popular theory that the introduction of Western culture led to the depopulation of Polynesian countries.^31^ Therefore, it was the white man’s mission to care for their survival. Second, medical education was closely connected to commercial interests. They believed that the ‘natives’ were best suited to cultivate the produce from the Pacific Islands. The lack of healthy labourers would increase the number of imported workers, who were considered less productive. This point was emphasised by quoting Dr John Cumpston, the Director of Public Health of Australia, who argued ‘the control of the island groups depends upon its efficient working population.’ More NMPs were beneficial to the imperial economy. Third, racial politics were taken into account. Quoting Cumpston again, Lambert argued that if diseases were not controlled, ‘the islands will become de-populated and then re-population must inevitably lead to international conflict.’ In other words, the creation of a larger medical school was ultimately intended to prevent ‘a Pacific War’.^32^ All involved administrations agreed that the extinction of the indigenous population and their subsequent replacement by the ‘Asiatics’ would disturb the international security of the Pacific.^33^ Exactly who the ‘Asiatics’ were is unclear from the documents.^34^ Considering that the idea of ‘Yellow Peril,’ a racialised warning against the growing East Asian influence in the Western world, was spreading at the time, it was likely a mixture of an acknowledgement of the expanding Japanese empire and the growing Chinese labour population in the Pacific. To summarise, the RF was persuaded by the school’s contribution to compensate for the British imperial history, economic value to the contemporary British Empire, and maintenance of future British influence in the Pacific world.

The RF funding was decided in 1926 by its International Health Board. This meant the school was considered a public health programme, not a full medical school, which would have been a concern for the previously approached Department of Medical Education. At the school’s establishment in 1928, the RF agreed to donate £2,000 on the condition that no less than £8,000 would be spent on building the school, with a training capacity of 40 students. The RF also shared the maintenance costs for the first four years in decreasing proportions: £5,445 (75%) in 1928, £4,356 (60%) in 1929, £3,267 (45%) in 1930, and £2,178 (30%) in 1931, out of the total annual budget of £7,260.^35^ A further £9,472 was offered for the concurrently established joint medical service to conduct centralised medical programmes in the WPHC territories. This was called the Maximum Rockefeller Scheme.^36^ Centralised education, treatment, and medical administration were essential to the scheme, which thus created the Western Pacific Health Service in 1928 under the direction of Lambert. It is on this basis that historian Annie Stuart evaluated the medical school as Lambert’s personal achievement, a Rockefeller project.^37^

While Lambert’s efforts to bring in the RF funding indeed contributed to the establishment of the CMS, it was not his solo effort that gave birth to the CMS. As an imperial institution, the establishment process was a multi-channelled initiative that created the grounds for intercolonial networks. The RF was a funding source of which the British took advantage. The Rockefeller funds were secured only after advancements were made with the Colonial War Memorial Hospital, the Tongan government’s actions, Western Samoa’s approach, and the WPHC’s plans for extending the NMP Service. Even if the RF did support the scheme, the WPHC planned for Fiji to establish the school regardless. Indeed, the WPHC had already been discussing the matter with the Colonial Office before the RF decision was made. Lambert himself admitted that the idea was ‘in the air and everyone had it just under the surface of their mind’.^38^

Financial support from the RF continued irregularly beyond the first four years of the original agreement. In 1934, the RF offered £2,200 to add a bacteriological laboratory, a pathological laboratory, and a post-mortem room to the CMS.^39^ After the funds proved insufficient for the new facilities, an additional £1,500 was transferred.^40^ The RF also donated used medical books and arranged a bibliographic donation from the New York Academy of Medicine to fill the CMS library.^41^ Considering that the RF spent nearly 45 million US dollars to establish the Peking Union Medical College in China, their contribution to the CMS was very small.^42^ Despite the amount being comparatively small, the RF contribution effectively reduced the financial burden on Fiji, which was already spending 10% of its total budget on medical purposes.^43^ Therefore, the CMS was a result of a collective effort between the WPHC, Tonga, Western Samoa, and the RF to supply medical staff to the British Empire in the South Pacific. Also, thanks to the existing educational infrastructure of the SMS, the Fijian indigenous healthcare system model was exported to neighbouring Pacific Islands.

## Making of an intercolonial institution

What makes the CMS such a significant historical case is that it was an ambitious imperial project designed for intercolonial cooperation between the territories of Britain, Australia, New Zealand, France, and the US. The Colonial Office had larger expectations for the CMS when the WPHC submitted the proposal for establishment in 1925. The British Secretary of State for the Colonies, Leo Amery, responded in August 1926 with some suggestions raised by the Colonial Advisory Medical and Sanitary Committee. The committee questioned its usefulness in comparison with successful colonial medical schools such as the Kitchener School of Medicine in Khartoum, Sudan, or the Ceylon Medical College in today’s Sri Lanka. In addition, the Colonial Office advised the WPHC to discuss the possibility of including its territories of Papua and New Guinea with Australia. Although the WPHC knew that Australia did not favour participation, they discussed the matter at the first International Pacific Health Conference held in Melbourne in December 1926.^44^

The main problem in bringing Australia into the initiative was that Australian authorities found it intolerable to subordinate themselves to another administration in any scheme. Despite being a settler colony itself, Australia was autonomous in decisions regarding its territories of Papua and New Guinea. It was different from New Zealand, which was generally more favourable to the British leadership. In fact, the International Pacific Health Conference was organised so that Australia could define Oceania as the ‘Austral-Pacific regional area’. Alexander Cameron-Smith has written that it was a framework within which Australia could unfold its ambitious medical imperialism. Another decisive factor was racism. The Australian government considered that Papuans and New Guineans did not have the intelligence to benefit from three to four years of higher education at the CMS.^45^ After it became clear that Australia would not join, the British Secretary of State approved the establishment of the CMS in principle in April 1927.^46^ The Australian attitude showed how the British Empire was not a monolithic entity. Australia was indeed an imperial power on its own. As a result, the CMS was initially founded by the WPHC, Tonga, and New Zealand territories as an intercolonial institution in the South Pacific.

Although Britain could not arbitrarily put Australia in the scheme, the CMS was a British-led project in a British colony, staffed by British officers who taught indigenous students across the British Empire in the Pacific. The composition of the CMS Advisory Board confirms the level of British influence in the intercolonial institution. The Chair was the Chief Medical Officer of Fiji, Dr Aubrey Montague. The second position was reserved for the Rockefeller Foundation, represented by Lambert. The remaining seats were taken by the Medical Superintendent of the Colonial War Memorial Hospital and the Secretary of the WPHC.^47^ In short, three seats on the Advisory Board, including the Chair, would be held by the WPHC staff based in Fiji. Only one place was reserved to acknowledge the donor, the RF. Although New Zealand and participating countries could join the Board meetings when they visited Fiji, the Board was not very inclusive.^48^ The majority of regular teaching staff, too, were British. The faculty comprised David Hoodless, the first principal of the CMS, who was the only full-time tutor, seven doctors, and two nurses. Exceptions were Lambert, who was an American, and one Fijian NMP.^49^

The CMS was operated by a collaboration between participant administrations. Fiji donated the land adjacent to the Colonial War Memorial Hospital for the construction of the CMS, which began in August 1927.^50^ Sir Eyre Hutson, the British High Commissioner for the Western Pacific, officially opened the CMS on 29 December 1928.^51^ The founding members consisted of British (including Tonga) and New Zealand territories, who signed an initial ten-year agreement for joint operation. All participating administrations shared the cost in proportion to the number of students they sent. At first, the annual cost per student was £170, regardless of whether each country filled its allocated quota or not.^52^ However, at the request of the Solomon Islands, unable to meet the quota, this policy changed. When an extra place occurred, the concerned administration could ask other countries to take up the unfilled quota. If no country was willing to send more students, the concerned administration would still pay for the running of the CMS but be exempt from paying for the students’ pocket money, rations, stores, clothing, books, and equipment.^53^

The intercolonial collaboration had its origins in the SMS period. The presence of Indian students at the SMS reflects Fiji’s historical connections to India. Indian students were mostly second- or third-generation Indian immigrants. The first Indian student graduated in 1926 to serve in the Indian districts of Fiji. The first overseas student was from Rotuma, a Fijian dependency, who graduated in 1912.^54^ Discussions about training NMPs for the Union Group began in 1911. Three students from the Union Group were fully funded as scholars by the Gilbert and Ellice Islands. Out of three, two students successfully graduated in 1916.^55^ The WPHC could also second NMPs to other territories upon request. The early cases of secondment became precedents of intercolonial NMP service, which gradually expanded in the following years. The intercolonial arrangement was convenient because the Governor of Fiji was also the High Commissioner for the Western Pacific.

Compared to its predecessor, the CMS expanded significantly both in size and diversity. The expansion enabled medical education to be accessible to many Pacific Islands. The CMS could accommodate 40 students at a time (i.e. an average of 10–13 per cohort), which was more than triple the capacity of twelve during the SMS period. At its inception, the CMS accepted students from the WPHC and New Zealand territories. The quota of each administration was allocated proportionately to its population. Fiji took up half of the student body, with 20 places for both Fijians and Indians. The Solomon Islands, the Gilbert and Ellice Islands, Tonga, and Western Samoa each sent four students. New Hebrides and the Cook Islands were allowed to send two students each. A few years later, Nauru and American Samoa joined the school to train some of their students.^56^

The admissions criteria reflected what the CMS expected from new students. Each country autonomously selected its scholars, provided they met three minimum requirements set by CMS: language, health, and age. The first required skill was English fluency. Since the British tutors taught in English, students had to be able to understand, read, speak, and write in English. Secondly, candidates had to be medically fit and vaccinated against smallpox, typhoid, and diphtheria. A strict medical test was introduced after the death of an NMP within one year of his graduation. Thirdly, students preferably had to be under twenty years of age, ideally between sixteen and eighteen.^57^ Occasionally, the CMS asked for additional signed consent from students to live in the dormitory, receive the provided diet, and conform with the school regulations.^58^

Racial heritage of the students was another crucial factor that determined their final acceptance to the CMS. Over the years, the Advisory Board developed a preference for NMPs ‘made from students nearly or entirely full-blooded natives’ instead of the European mixed-race ‘half-caste’ students. The ‘half-caste’ students were deemed not native enough to follow the CMS policy of preserving indigenous customs.^59^ Students even had to submit their lineage information that contained the race of their family tree. It was only when countries such as the Solomon Islands could not find suitable candidates that the CMS accepted ‘half-caste’ candidates.^60^ Owing to this racial policy, the CMS could boast that their graduates had ‘pure native blood’.^61^

Each country selected its own scholars who met the criteria to enter the CMS. Fiji held competitive entrance exams to pick the best applicants. An established reputation of 40 years of NMP history probably contributed to this popularity. Similarly, recruitment was comparatively easy in Tonga. According to Heather Leslie Young, Tonga sent the top two students from its two high schools in 1928.^62^ The CMS annual report detailed that the students were selected through a scholarship examination in which the top-ranked student was entitled to study in Australia, while the second- and third-ranked students were offered training at the CMS.^63^ The Gilbert and Ellice Islands found students from what was considered a good secondary school in Ocean Island. In the first ten years of the CMS, three Ellicean and ten Gilbertese students graduated. Competent marks on admission did not always lead to good final grades. However, the CMS still let underachieving students graduate because they thought lower-standard NMPs were better than none at all.^64^ Such a compromise could only be made for students who fulfilled the minimum standard for graduation.

In contrast, colonial authorities in the Solomon Islands considered it hard to find candidates for the CMS. Generally, there was strong racial prejudice against the Solomon Islanders, who were categorised as Melanesians and considered to be inferior to other Pacific Islanders. Richard Kane, the Resident Commissioner of the Solomon Islands, reported that the Solomon Islanders are not ‘lacking in brain’, but he doubted whether they could make correct medical judgments.^65^ Believing that delays in NMP training would worsen the health conditions in what he called the ‘retrograde islands’, Lambert suggested funding Fijian students to serve in the Solomon Islands instead.^66^ This suggestion was rejected for fear of losing highly valued Fijian NMPs through illness while working in the Solomons in what was perceived as a poor climate.^67^ As an alternative, a batch of Solomon Islanders was sent to the Queen Victoria School, Fiji, for preliminary education on Fiji’s standards before applying to the CMS.^68^ At the time, the Solomon Islands government largely relied on missionaries to run secondary schools. The first government school in the Solomon Islands was only established in 1948 following the establishment of the Protectorate Education Department in 1946.^69^ Later, the Solomon Islands government found students to attend the CMS. One of them was George Bogese, a graduate of the Melanesian Mission School, who was recommended by the District Officer of Gizo.^70^ He was banned from the CMS dormitories for misconduct but managed to graduate and became the first NMP of the Solomon Islands.^71^

Things were considered even more difficult in the New Hebrides, an Anglo-French Condominium. To begin with, gaining French consent was a challenge. The British, therefore, took a cautious approach in negotiations, believing that the French authorities cared little for indigenous welfare. They reported that the eventual French agreement to be part of the CMS was a diplomatic decision, considering their relationship with Britain.^72^ As for students, the New Hebrides government considered three potential sources for recruitment: educated chiefs, recommendations from mission teachers, and former French vessel cargo workers. However, it was reported that the chiefs would not leave the islands. The French authorities disapproved of the suggestion of the mission teachers. Cargo workers were considered unsuitable for a medical career. In the end, New Hebrides compromised to pay for Fijian students who agreed to work in New Hebrides after graduation. It was not until 1943 that the first New Hebrides student graduated from the CMS.^73^

New Zealand territories showed a higher level of secondary education. Western Samoa selected students among medical cadets who worked at the general hospital in Apia. They were reportedly more mature, medically knowledgeable, and familiar with hospital duties. Naturally, they made good medical students. The Cook Islands chose students from their existing scholars at Te Aute College, a Maori school in New Zealand.^74^ Tokelau already had NMPs from the SMS period. Niue only sent a student much later who graduated in 1950.^75^ New Zealand territories’ selection process was centrally managed in Wellington. They first collected recommendations, interviewed candidates, and then considered distributing candidates’ expertise in the colonial public service more broadly. Generally, students from New Zealand territories were academically competent at the CMS. For example, the Cook Islanders received an average of six awards per student, and Western Samoans won 5.4 awards per student. It was a remarkable rate when all other administrations together received an average of 2.4 awards per student. In total, 37.2% of the awards given from 1930 to 1938 went to students from the Cook Islands and Western Samoa.^76^

Nauru, not among the founding members of the CMS, began sending students in 1934.^77^ It is not clear why Australia allowed Nauru to join. When the WPHC proposed the CMS, Australia did not join it so that it could maintain full control of its dependencies. Nauru was, in effect, an Australian colony, but officially, it was a co-trusted League of Nations mandate between Britain, Australia, and New Zealand. Therefore, it was still part of the British sphere of influence. In addition, Nauruans were categorised as Micronesians, not Melanesians, whom Australia considered not intelligent enough to be educated. Moreover, the small population of about 1,500 would only require three NMPs for the entire country. This was financially affordable compared to much more densely populated Papua or New Guinea.^78^ Acknowledging the needs of the small country, the CMS waived the usual joint administrative fee of £170 per student.^79^ Nauru only paid £100 per student for the duration of training three NMPs. As a result, two former orderlies and one former dispenser at the Administration Hospital of Nauru trained at the CMS.^80^

The addition of American Samoa in 1933 excited the British authorities very much.^81^ The French empire was represented through New Hebrides, but it was part of the British Empire as well. American participation could really raise the profile of the CMS to become an inter-imperial school. Contrary to the British hopes, the excitement quickly turned into complications with the American attitude. The admissions request for American Samoa was made in a short and rushed telegraph. The CMS had no clue how American Samoan students were selected, nor did they verify that the candidates met the required educational standards.^82^ The result was the first American Samoan ranking at the bottom of his year’s cohort. Inconsistent attitudes by American authorities caused further British annoyance. In 1933, the year American Samoa joined the CMS, the Public Health Officer of American Samoa severely criticised the NMP Service in his annual report. Nonetheless, the US Navy, in charge of administering American Samoa, continued to make urgent requests. They abruptly withdrew the students a few years later, only to ask the CMS for readmission in another few years.^83^ This short-lived withdrawal was to train the students more quickly in Guam for immediate service during the Second World War.^84^ To follow the Colonial Office policy promoting Anglo-American cooperation in the Pacific, the CMS reluctantly accepted American Samoa.^85^ Together with Nauru, New Hebrides, and Tonga, the presence of countries like American Samoa added a stronger intercolonial character to the CMS, which was fundamentally different from other medical schools made for one particular country. The CMS was imperial in that the intent was to expand its influence as much as possible. It succeeded in reaching beyond the jurisdiction of the British Colonial Office, yet it served to benefit the British Empire.

## Medical curriculum for British colonies

An examination of the medical curriculum at the CMS allows an understanding of what the participating empires expected from indigenous doctors. The curriculum of the CMS was much more developed compared to its predecessor, the SMS. The SMS offered practice-based training to its students. Students trained for three years at the Colonial Hospital in Suva, where everyone boarded at an adjoining hostel. Notably, there was no real curriculum at the SMS. On weekday mornings, students attended a one-hour lecture by the Chief Medical Officer or Senior Medical Officer of Fiji. Anatomy, Physiology, Medicine, and *Materia Medica* were taught without a fixed syllabus. The textbooks were in English, but teaching was done in Fijian because students were not proficient in English. After the lecture, the rest of the day was spent working at the Colonial Hospital. Everyday duties included dressings, washing patients, seeing outpatients, rounding the wards, observing operations, carrying patients, assisting post-mortems, and tending food gardens. Each student was assigned to indigenous male patients admitted during their days on duty. For the female wards and Indian wards, which were separated, the students served on a rotation basis. Students were also put on standby for any ambulance emergencies and were responsible for monitoring delirious or insane patients.^86^ Patients diagnosed with lunacy at the Colonial Hospital were eventually transferred to Suva’s Public Lunatic Asylum (later St. Gile’s Psychiatric Hospital).^87^ In short, student life at the SMS was more akin to that of unpaid medical orderlies than full-time medical students.

Medical education at the CMS was a version of British imperial education. This was essential for an intercolonial school, where the participating countries had to reach a consensus on the expected outcome of their invested education. The key aspect of it was to teach the same kind of Western medicine to students from different backgrounds. Through the CMS and its graduates, the British implanted Western medicine in the Pacific Islands. For this reason, David Brewster, the former dean of the Fiji School of Medicine, felt that the introduction of Western biomedicine was the main historical contribution of the CMS.^88^

Also, the CMS had an explicit syllabus for each stage of education that was designed to be in line with medical schools in other colonies of the British Empire. When it was established in 1928, the Colonial Office consulted regarding the curriculum with the principals of the Medical School at Mulago, Uganda, and the College of Medicine, Singapore, to assess whether it kept up with the imperial norm. Both principals returned stark criticisms on three main points: the insufficiency of a three-year curriculum, the lack of practical training, and the absence of Biology in the coursework. Cases outside the British Empire were also considered, noticing the impact of medical schools in French West Africa.^89^ The Colonial Office even arranged a tour of colonial medical schools for Hoodless, the principal of the CMS, later in 1939. The plan was to visit the King Edward VII Medical College in Singapore, the Ceylon Medical School in Colombo, the Makerere Medical School in Uganda, the Kitchener School of Medicine in Sudan, and a French Medical College in Dakar.^90^ Although the outbreak of the Second World War interrupted the itinerary, Hoodless had still been able to visit Africa.^91^

Following the discussions with the Colonial Office and other medical schools, Hoodless restructured the curriculum. Beginning with the cohort enrolled in 1931, the CMS provided a four-year education, which was a change from the previous three-year course.^92^ The new four-year curriculum consisted of two stages, including both theoretical and clinical education. During the junior period, which lasted one and a half years, students received full-day lectures in Physics, Biology, Anatomy, and Physiology. This was quite a shift from the SMS, which only spared one hour each day for lectures. Clinical tasks were systematically integrated into the curriculum for advanced students. Senior students spent two and a half years engaging in clinical work during the mornings and attending lectures in the afternoons. For clinical work, students attended the medical wards, surgical wards, female wards, European wards, hospital dispensaries, and outpatients.^93^ Field visits to nearby villages for the Hygiene course emphasised practical training.^94^ What was similar to the SMS was that students made a substantial labour contribution at the Colonial War Memorial Hospital, to which the CMS was attached. This labour contribution was important to the extent that the colonial government became concerned about the lack of hospital staff when the student numbers at the CMS were not at their maximum level.^95^ Through the CMS, Fiji enjoyed more dressers and clinicians at their disposal without paying salaries other than scholarships.

Admission to the CMS did not guarantee graduation. Many students struggled to adjust to both academic and social life. Failing final exams was not uncommon. When a failure occurred, students were given a second chance or even a third chance if they were diligent. Repeated failures could result in the cancellation of their studentships.^96^ A notable pattern of misconduct was gendered violence. On one occasion, a student was dismissed for attacking another student and showing ‘gross impertinence’ to European nurses.^97^ Another student was immediately expelled after he stroked the supervising nurse’s cheek with his hand and pushed a fellow student who tried to stop him.^98^ Yet another student was dismissed for causing a ‘very serious injury’ to an indigenous obstetric nursing student after enticing her into his dormitory room and keeping the door locked for twenty minutes.^99^ Only students who met both academic and disciplinary standards could qualify as an NMP.

**Figure 2 and Figure 3.**
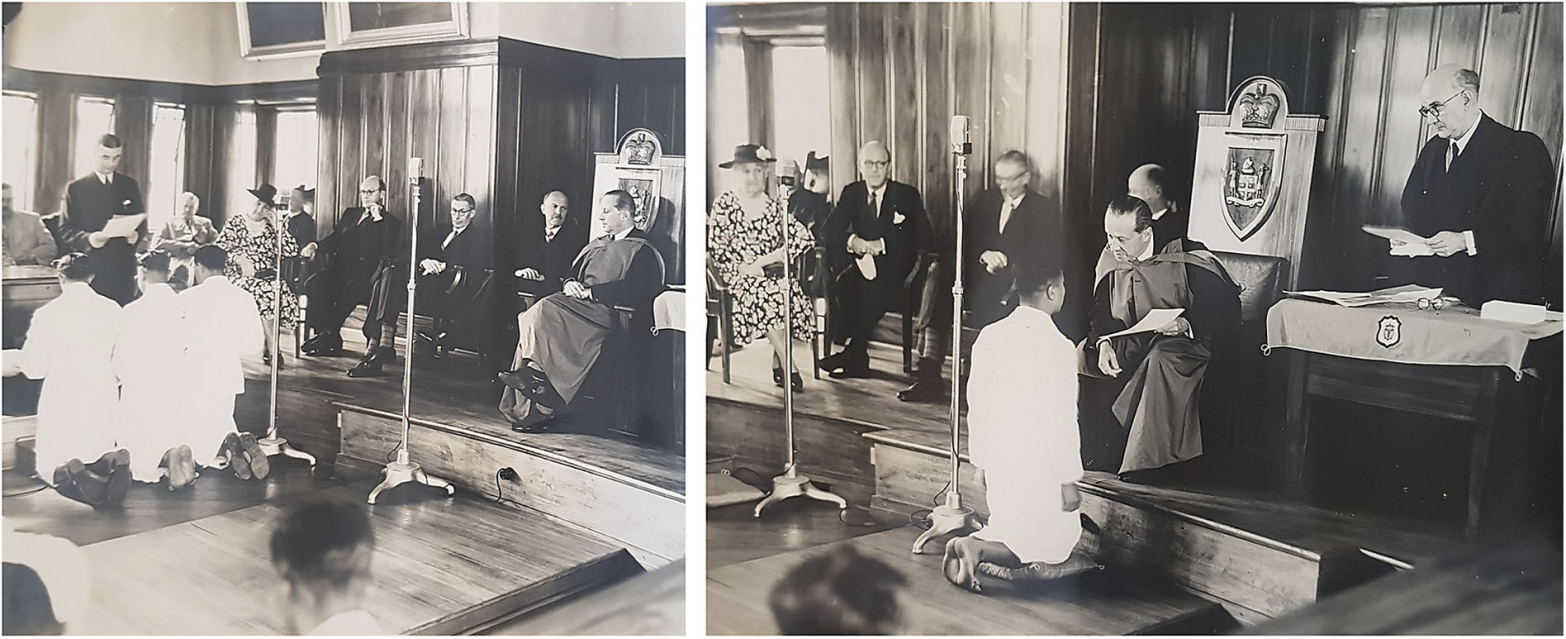
Graduation ceremony in the Legislative Council Chamber. Left: Graduates are taking the Hippocratic Oath. Right: Sir Harry Luke is presenting the diploma in his DLitt robe. [Margaret Guthrie’s Private Collection].

Another point that affirms the CMS followed the imperial standards of medical education is the introduction of a European-styled graduation ceremony. Its first graduation ceremony took place in 1939 when students from five different Pacific Islands knelt to take the Hippocratic Oath, which was administered by the Acting Director of Medical Services of Fiji. As shown in Figure 2 and Figure 3, the graduates went on their knees again to receive their NMP diplomas from the High Commissioner for the Western Pacific, Sir Harry Luke. Luke was dressed in his scarlet Oxford DLitt robe, which he supposed was never seen before in the Pacific Islands. It was Luke’s idea to hold the graduation ceremony ‘in proper surroundings on a proper scale, and be made as far as possible an impressive academic affair’. For Luke, it was an opportunity to showcase the achievements of the CMS, which he believed were ‘the most beneficent gifts which British rule has conferred upon the native races of the Pacific’.^100^

The graduation ceremony was an imperial ritual. Luke held the function at the newly built Legislative Council Chamber, one of the most important buildings owned by the colonial government. Doctors, dentists, European nurses, indigenous nurses, friends and families of the graduates, and all students from the CMS were present at the ceremony. In addition, Executive Council members, Legislative Council members, WPHC staff, and a number of prominent citizens were invited to take part in the colonial spectacle. With the exception of American Samoan students, the audience represented people from across the British Empire, comprising Britain, Fiji, Rotuma, the Solomon Islands, the Gilbert and Ellice Islands, Tonga, Western Samoa, the Cook Islands, Nauru, and India.^101^ Students were shown to the audience taking the oath and kneeling to the High Commissioner after learning Western medicine from British tutors in English with peers from other colonies.

## Colonial encounters through a medical career

The intercolonial operation of the CMS facilitated various forms of colonial encounters. As Luke stated, the CMS was ‘a microcosm of the Pacific’ where the students benefited from ‘mingling with other races of the Pacific and with Indian fellow-students’.^102^ In 1938, there were reportedly four races, eleven different languages, and seven religions represented among the students.^103^ Such diversity allowed its governing bodies to easily test the existing racial notions of the Pacific Islanders. The CMS was, in that sense, a laboratory for racial science that had samples from across the Pacific in a limited space for a designated period of time.

From the British perspective, there were three races in Oceania: Polynesians, Melanesians, and Micronesians. Polynesians were the most admired among the three groups of people. As Maile Arvin wrote, white Europeans continuously described Polynesians as almost white, and they were popularly suspected to be of Aryan descent. On the contrary, Melanesians were the least preferred for their darker pigment and were often subject to racial prejudice that was similar to what was expressed towards other ‘Black’ people, such as Africans.^104^ They were called the *Oceanic Negroes.* Micronesians were similarly racialised with association to Southeast Asians, being positioned somewhere between Polynesians and Melanesians in the European preference.^105^

At the CMS, racial stereotypes were established through an ancient humoral language. The majority of the students were described as ‘easy-going sanguine type, typical South Seas Islanders’ with the exceptions of ‘phlegmatic’ Solomon Islanders and ‘choleric’ Gilbertese students.^106^ Student assessments were always framed in racial categories, too. For example, Western Samoa and the Cook Islands exceeded Melanesia in terms of ‘mental gifts’. Students from the Gilbert and Ellice Islands, who were Micronesians, were also considered mentally superior to Fijians, who were Melanesians. Even among the Melanesian countries, the Solomon Islands and New Hebrides students were placed at the bottom of the perceived intellectual hierarchy.^107^ Such racial understanding can be confirmed by preliminary secondary education in Fiji, which is only offered to students from the Solomon Islands and New Hebrides.^108^

Despite efforts to rank and stereotype the Pacific races, it did not take long for the CMS to learn that the Pacific Islanders were far more intelligent than suggested by the general European perception. They were surprised that Indians, presumably of a superior civilisation, were not among the brightest students at the CMS.^109^ While the Europeans still believed Polynesians were more intelligent than Melanesians, the CMS authorities concluded that student performance was determined more by the educational system of their country of origin than by the intrinsic intellectual abilities of each race.^110^ It is significant that this conclusion came in the 1930s when many Europeans believed that race determined one’s intellectual abilities.

The intercolonial environment of the CMS allowed some students to enjoy showing off their newly acquired cosmopolitan tastes. There was an apparent craze for shopping for European attire and items before leaving Fiji. One student shocked the tutors by choosing to appear in full European dress immediately after graduation. This was surprising because students were required to wear their traditional dress while at the CMS.^111^ Those who did not have enough money to go shopping in Suva before returning to their countries asked the WPHC for a cash advance on their salaries. To receive cash advances, the CMS imposed a condition of submitting a written application with details of the expenditure plan.^112^ The condition was added after a student purchased two dozen shirts with his advanced salary instead of the medical equipment that he orally told the WPHC he would buy: a stethoscope, a microscope, and a benzine lamp.^113^ The most popular items were shirts, shorts, trousers, sulus (a type of traditional clothing), blazers, and European coats. They also bought wooden boxes, irons, umbrellas, blankets, and picture frames to take back to their home islands.^114^

The CMS wanted students to be ‘native’ but not too native. Given the students’ desire to dress in European style, it may seem like the CMS promoted Westernisation. While the CMS expected graduates to deliver Western medicine to remote islands where European officers would not visit, the CMS taught the students to ‘work as a native amongst natives without ideas beyond his station.’^115^ This philosophy can be reaffirmed in the concern of Sir Alexander Grantham, the High Commissioner for the Western Pacific, that American Samoan students sent to Guam would be ‘de-nativised, but yet will not be accepted as the equal of white Americans’.^116^ It was a tool to avoid spoiling the students with a comfortable lifestyle and to differentiate them from European doctors. Contradictorily, the age requirement for admissions was put in place to select young students ‘before they have become completely set in the native mental mould’.^117^

Socially, graduate NMPs were asked to abstain from interfering in provincial affairs unrelated to medical practice.^118^ This measure may have been set for ethical reasons, but it also limited the NMPs to the specialist sphere of medicine. Considering their elite status, with many born into privileged families, they could potentially become more influential than the colonial government wanted them to be. Most NMPs followed this guidance, yet their very presence already meant more than medicine. As Alexandra Widmer argued, NMPs were administrators of the colonial state, demonstrating what she called a ‘nascent biomedical citizenship’.^119^ Jacqueline Leckie also noted that NMPs were not only intermediaries of Western medicine but also representatives of colonial authority.^120^

Ultimately, the apparent limitation of CMS was its inability to break through the institutionalised racism of the colonial South Pacific. ‘Who’s the boss?’ questioned the Public Health Officer of American Samoa when he criticised the NMP Service for risking insubordination among Pacific Islanders.^121^ The British, too, did not desire such a thing. By defining the NMP Service as a ‘subordinate medical service’, racial segregation could be maintained in the British territories.^122^ Basically, European doctors treated Europeans, administered hospitals, and controlled quarantine. The NMPs were asked to care for indigenous health except for European emergencies. It was clear from the early years that the CMS would not become a full-fledged medical college granting medical degrees. Subsequently, the NMP Service could not become a regular medical service because the NMPs held inferior diplomas.^123^ The colonial authorities wanted NMPs to remain under European supervision to prevent their medical practice and behaviour from deteriorating.^124^ Rebellious NMPs could be dismissed. For example, NMP Tabonteren of the Gilbert and Ellice Islands got his licence revoked for inadequate work performance and disobedience. This included him choosing to wear ‘complete European dress’ and refusing to wear the uniform in traditional dress.^125^ Publication and circulation of the *Native Medical Practitioner*, a semi-annual journal first published in 1930, was one of the ways to govern the NMPs, as its purpose was to make the NMPs ‘coherent and articulate’.^126^

## Conclusion

After ten years of operation, the British Secretary of State for the Colonies introduced the CMS as ‘specially successful’ to the Parliament.^127^ Using the case of the CMS, the Colonial Office argued that colonial healthcare should eventually be achieved through ‘progressive training of medical staffs drawn from the local inhabitants’ instead of increasing the Colonial Medical Service.^128^ In its first ten years, the CMS produced 209 NMPs, of whom 51 were from the Pacific Islands outside Fiji.^129^ It expanded to include a dental training course in 1945. In the 1950s, the CMS revised its curriculum to offer a five-year course that qualified its graduates as Assistant Medical Practitioners. Niue, Papua New Guinea, and the United States Trust Territories started to send students to the CMS during this period. In 1961, the CMS rebranded itself as the Fiji School of Medicine and is now incorporated into Fiji National University.^130^

The CMS case shows how medical education in the early twentieth century South Pacific was an imperial project. The British established the CMS for population control, labour resource management, and racial politics in the Pacific. The strategic importance of the CMS can be highlighted by the fact that medical schools were not founded until after the Second World War in other small islands of the British Empire, such as in the Caribbean or the Indian Ocean. As an intercolonial institution, significant developments were made in the CMS curriculum to meet the imperial norm. After consulting with medical schools in other British colonies, the CMS syllabus was revised into a four-year curriculum with both theory and practical training. Owing to its intercolonial nature, the CMS became one of the few spaces in Oceania where colonial encounters happened on such a large scale. The student body of the CMS was an early case of a ‘Pacific community’. NMPs, who spent their youth in the ‘microcosm of the Pacific’, later took part in promoting regionalism in the Pacific. Many of them were present at the South Seas Conference in 1947, which resulted in the creation of the South Pacific Commission.^131^ Also, significantly, colonial authorities learned through the CMS that race did not determine one’s intellectual abilities. This wisdom was gained by the students, too. As Ratu Sir Kamisese Mara, who became a long-time political leader after Fiji’s independence, remarked in his memoirs, he learned ‘the most valuable knowledge that under the skin all people are the same’ during his time as a student at the CMS.^132^ It was founded for colonial governance, but ironically, it became a space for discovering equalities.

## Footnotes

1 This article is part of an unpublished DPhil thesis. Parts of this article using the primary sources from the Rockefeller Archive Center have been discussed in Hohee Cho, ‘The Establishment of the Central Medical School’, a research report for the Rockefeller Archive Center, 2020.

2 David Brewster’s book offers a good overview of the history of medical education in Fiji: *The Turtle and the Caduceus: How Pacific Politics and Modern Medicine Shaped the Medical School in Fiji, 1885–2010* (Bloomington, United States of America: Xlibris Corporation, 2010). Margaret W. Guthrie wrote a biographical account of the first principal of the CMS in *Misi Utu: Dr. D.W. Hoodless and the Development of Medical Education in the South Pacific.* (Suva, Institute of Pacific Studies: University of the South Pacific in Association with the South Pacific Social Sciences Association, 1979). Annie Stuart studied the establishment of the CMS with a strong focus on the Rockefeller Foundation involvement in ‘Parasites Lost? The Rockefeller Foundation and the Expansion of Health services in the Colonial South Pacific, 1916–1939’, (unpublished PhD thesis: University of Canterbury, 2002). Two of her papers pay attention to the identity of Native Medical Practitioners: ‘Contradictions and Complexities in an Indigenous Medical Service: The Case of Mesulame Taveta’, *The Journal of Pacific History*, 41, 2 (2006), 125–43, and ‘We Are All Hybrid Here: The Rockefeller Foundation, Sylvester Lambert, and Health Work in the Colonial South Pacific’, *Health and History,* 8, 1 (2006), 56–79. Other works that touch upon the history of the CMS include Alexandra Widmer’s ‘Native Medical Practitioners, Temporality, and Nascent Biomedical Citizenship in the New Hebrides’, *PoLAR: Political and Legal Anthropology Review,* 33 (2010), 57–80; and Alexander Cameron-Smith’s book, *A Doctor across Borders: Raphael Cilento and Public Health from Empire to the United Nations* (Canberra: ANU Press, 2019). Greg Fry notes the role of the CMS in regional cooperation in ‘The South Pacific “Experiment”: Reflections on the Origins of Regional Identity’, *The Journal of Pacific History*, 32, 2 (1997), 180–202, and in *Framing the Islands: Power and Diplomatic Agency in Pacific Regionalism* (Canberra: ANU Press, 2019).

3 Ilai Motonicocoka, ‘The Story of *Lila Balavu* (Wasting Sickness) and of the *Cokadra* (Dysentery); and the *meke* (ballads) relating to those events’, Appendix 1, *Report of the Commission Appointed to Inquire into the Decrease of the Native Population* (Suva: Edward John March Government Printer, 1896).

4

Brij V. Lal , Chalo Jahaji: On a Journey through Indenture in Fiji. (Canberra: ANU Press, 2012), 143–5.

5

David Hoodless , Central Medical School, Suva, Fiji (Suva: Government Press, 1947), 3 .

6 Plan of Training of Native Medical Practitioners at the Colonial Hospital Suva, 25 July 1919, WPHC4/IV 1326/1919, Western Pacific Archives. MSS & Archives 2003/1. Special Collections, University of Auckland Libraries and Learning Services (WPA).

7 Rules made by the Chief Medical Officer under section 10 of Ordinance No.11 of 1888, Governing the Conduct of Native Medical Practitioners, WPHC4/IV 1326/1919, WPA.

8 Hoodless, *op. cit.* (note 5), 4.

9 S.M. Lambert, Medical Care of South Sea Natives, 12 October 1923, RF RG1.1 S419 B1 F5, Rockefeller Archive Center (RAC).

10

Sandra Tomkins , ‘The Influenza Epidemic of 1918–19 in Western Samoa’, The Journal of Pacific History, 27, 2 (1992), 181–97.

11
*Native Medical Practitioner,* 3, 1 (1939), WPHC4/IV 438/1934, WPA.

12 Legislative Council, Council Paper No.40 Annual Medical and Health Report, Medical Department. 1938. National Archives of Fiji.

13

Seth Archer , Sharks Upon the Land: Colonialism, Indigenous Health, and Culture in Hawai‘i, 1778–1855 (Cambridge: Cambridge University Press, 2018), 1–3, 240–245.

14 From C. H. Rodwell to Lambert, 29 September 1923, RF RG1.1 S419 B1 F5, RAC.

15

Norma McArthur , Island Populations of the Pacific (Canberra: ANU Press, 1967), 35 .

16 There are many ways to describe the Indian descendant population in Fiji: Indians, Fijian-Indians, Indo-Fijians, and Fiji-born Indians. All these names have been subject to political debates and carry specific historical connotations. This article follows the historical use of racial categories (‘Indians’ or ‘Fijians’) and does not intend to apply the same term to contemporary peoples.

17 Chief Medical Officer, Fiji Medical School for Medical Students, 5 February 1924, RF RG1.1 S419 B1 F6, RAC.

18

S.M. Lambert , The Depopulation of Pacific Races (Honolulu: The Museum, 1934), 24–25 .

19 Enclosure No.1 of Council Paper No.56 Central Medical School, 1926. WPHC4/IV 3569/1926, WPA.

20
*Ibid.*

21 From George S. Richardson, the Administrator of Western Samoa to Dr. Victor G. Heiser, 3 September 1924, RF RG1.1 S419 B1 F6, RAC.

22 Victor Heiser, 11 November 1924, RF RG1.1 S419 B1 F6, RAC.

23 1^st^ Annual Report, Western Pacific Health Service 1928. F1977 419 Western Pacific Health Service Annual Report, 1928, RG5 Rockefeller Foundation Records, International Health Board/Division Records, RAC.

24 From the Premier to the British Agent and Consul, 7 February 1925, RF RG1.1 S419 B1 F6, RAC.

25 Enclosure No. 1 of Council Paper No. 56 Central Medical School, 1926. WPHC4/IV 3569/1926, WPA.

26 Asks to be Furnished with Information Concerning the Native Medical School at Suva. From: Governor General of New Zealand, 25 November 1925, WPHC4/IV 2856/1925, WPA.

27 Decisions given by the Hon. Minister of External Affairs Concerning Matters Brought Forward by the Administrator of Western Samoa, December 1925, ACGA 8280 EX8/24 Part.1 R17962755, Archives New Zealand Te Rua Mahara o te Kāwanatanga, Wellington (ANZ).

28 Stuart, Parasites Lost*, op. cit.* (note 2), 30–48.

29 Council Paper No. 56 Central Medical School, 1926. WPHC4/IV 3569/1926, WPA.

30 Enclosure No. 2 of Council Paper No. 11 Central Medical School, 1927. WPHC4/IV 3569/1926, WPA.

31

Basil Thomson , The Fijians: A Study of the Decay of Custom (London: William Heinemann, 1908), vii–xix .

32 These justifications are found in S.M. Lambert, Medical Care of South Sea Natives, 12 October 1923, RF RG1.1 S419 B1 F5, RAC.

33 S.M. Lambert, The South Pacific, 30 July 1926, RF RG1.1 S419 B1 F7, RAC.

34 The label “-tics” is usually ascribed to an alien external system, generalising the entire subject system without recognising individuality or diversity. Kenneth L. Pike , Language in Relation to a Unified Theory of the Structure of Human Behavior (Berlin, Boston: De Gruyter Mouton, 2015), 37–9.

35 Minutes of the International Health Board, 4 November 1926, RF RG1.1 S419 B1 F5, RAC.

36 Council Paper No. 11 Central Medical School, 1927. WPHC4/IV 3569/1926, WPA.

37 Stuart, Parasites Lost*, op. cit.* (note 2), 223–53.

38 From Lambert to International Health Board, 20 July 1924, RF RG1.1 S419 B1 F6, RAC.

39 Minutes of Proceedings of a Meeting of the Central Medical School Advisory Board, 13 November 1934, WPHC4/IV 103/1934, WPA.

40 Minutes of the Rockefeller Foundation, 1–2 November 1935, RF RG1.1 S419 B1 F5, RAC.

41 From Acting Chief Medical Officer to Lambert, 28 November 1934, RF RG1.1 S419 B1 F10, RAC.

42

M. Margorie King , ‘The Social Service Department Archives: Peking Union Medical College 1928–1951’, American Archivist, 59, 3 (1996), 341 10.17723/aarc.59.3.e80325656688j02211619522.

43 Council Paper No. 56 Central Medical School, 1926. WPHC4/IV 3569/1926, WPA.

44 From the Secretary of State to the Governor of Fiji, 28 August 1926, Council Paper No. 56 Central Medical School, 1926. WPHC4/IV 3569/1926, WPA.

45 Cameron-Smith, *op. cit.* (note 2), 93–132.

46 From Colonial Secretary’s Office to the Western Pacific High Commission, 11 April 1927, WPHC4/IV 3569/1926, WPA.

47 From the Colonial Secretary to the Acting Secretary of Western Pacific High Commission, 30 August 1928, WPHC4/IV 2562/1928, WPA.

48 Minutes of a Meeting of the Central Medical School Advisory Board, 10 July 1928, ACGA 8280 EX8/24 Part.1 R17962755, ANZ.

49 1^st^ Annual Report, Western Pacific Health Service 1928, RF RG5 S3.419 B161 F1977, RAC.

50 From Lambert to Heiser, 31 August 1927, RF RG1.1 S419 B1 F7, RAC.

51 1^st^ Annual Report, Western Pacific Health Service 1928, RF RG5 S3.419 B161 F1977, RAC.

52 H.H. Rushton, Memorandum for Central Medical School, Suva, 26 September 1928, WPHC4/IV 2868/1928, WPA.

53 Unfilled Studentships Central Medical School, 19 March 1929, WPHC4/IV 2868/1928, WPA.

54 Brewster, *op. cit.* (note 2), 408–9.

55 Native Medical Students, Union Group.WPHC4/IV 1801/1911, WPA.

56 1^st^ Annual Report, Western Pacific Health Service 1928, RF RG5 S3.419 B161 F1977, RAC.

57 Central Medical School, Suva, Fiji, Annual Report for 1931. RF RG5 S3.419 B162 F1983, RAC; From Acting Colonial Secretary to the Prime Minister, 20 September 1935. A518 X832/1/2 Part.1, National Archives of Australia (NAA); From Acting Colonial Secretary to the Prime Minister of Australia, 14 September 1938, A518 X832/1/2 Part.1, NAA.

58 Admission of Student into Central Medical School, Suva, 18 April 1935. A518 X832/1/2 Part.1, NAA.

59 Memorandum to Participating Groups, 21 February 1935, ACGA 8280 EX8/24 Part.1 R17962755, ANZ.

60 Minutes of Proceedings of a Meeting of the Central Medical School Advisory Board, 13 November 1934, WPHC4/IV 103/1934, WPA.

61 Thomas Clunie, Abstract from *Native Medical Practitioner*, Tropical Disease Bulletin, November 1936, RF RG5 S3.417 B161 F1975, RAC.

62 Heather Leslie Young, ‘Tongan Doctors and a Critical Medical Ethnography’, *Anthropological Forum,* 13, 3, 278.

63 Central Medical School, Suva, Fiji. Annual Report for 1931. RF RG5 S3.419 B162 F1983, RAC.

64 Minute, 10 June 1938, WPHC4/IV 1584/1937, WPA.

65 From the Resident Commissioner to the High Commissioner, 19 May 1927, WPHC4/IV 3569/1926, WPA.

66 From the Resident Commissioner to the High Commissioner, 23 November 1927, WPHC4/IV 3569/1926, WPA.

67 With Reference to Recruits to Medical School from Melanesia, 20 June 1927, WPHC4/IV 3569/1926, WPA.

68 From Assistant Director of Education to the Secretary, 27 September 1927, WPHC4/IV 3569/1926, WPA.

69

Karen Ann Watson-Gegeo and David Welchman Gegeo , ‘Schooling, Knowledge, and Power: Social Transformation in the Solomon Islands’, Anthropology & Education Quarterly, 23, 1 (1992), 10–29 ; Clive Moore and Simeon Bouro, Chapter 18 Education, *Solomon Islands in Pictures* (Online Publication: University of Queensland, 2017) https://law.uq.edu.au/research/uqsip/solomon-islandsin-pictures.

70

Hugh Laracy , Watriama and Co: Further Pacific Islands Portraits (Canberra: ANU Press, 2013), 234 .

71 Progress of students at Central Medical School: Asks if half-yearly reports could be furnished. WPHC4/IV 609/1929, WPA.

72 Central Medical School: Participation of New Hebrides in, and financial contribution to. WPHC4/IV 29/1929, WPA.

73 Brewster, *op. cit.* (note 2), 409.

74 Central Medical School, Suva, Fiji. Annual Report for 1931. RF RG5 S3.419 B162 F1983, RAC.

75
*History and Graduates of the Medical School known as Suva Medical School, 1888–1927, Central Medical School, 1928–1960, Fiji School of Medicine, 1961–present* (Suva, 1979).

76
*Native Medical Practitioner,* 3, 1 (1939), WPHC4/IV 438/1934, WPA, 452.

77 From the Acting Colonial Secretary to the Prime Minister of Australia, A518 X832/1/2 Part.1, NAA.

78
*Native Medical Practitioner,* 3, 1 (1939), WPHC4/IV 438/1934, WPA, 456.

79 Legislative Council, Council Paper No. 40 Annual Medical and Health Report, Medical Department. 1938, NAF, 31.

80 From the Administrator to the Prime Minister’s Department, 4 July 1935, A518 X832/1/2 Part.1, NAA.

81
*Native Medical Practitioner,* 3, 2 (1939), WPHC4/IV 438/1934, WPA, 465.

82 Council Paper No. 54, Medical Department, 1934. ACGA 8280 EX8/24 Part.1 R17962755, ANZ, 10.

83 From the High Commissioner to the Secretary of State for Colonies, 31 January 1945, CO 83/241/10, The National Archives of the UK (TNA).

84 Suggested Establishment of Medical Centers in Forward Areas, 19 August 1944, File No S-37, General Administrative Files, ca. 1944–1951, Record Group 313, National Archives at College Park, College Park, MD.

85 Letter to Mr. Sidebotham, 3 December 1945, CO 83/241/6, TNA.

86 Plan of Training of Native Medical Practitioners at the Colonial Hospital Suva, 25 July 1919, WPHC4/IV 1326/1919, WPA.

87 For the history of psychiatric care in Fiji, see: Jacqueline Leckie , Colonizing Madness: Asylum and Community in Fiji (Honolulu: University of Hawai‘i Press, 2020).

88 Brewster, *op. cit.* (note 2), 219–26.

89 Correspondence on the Subjects of the Syllabus and the Four-Year Course, ACGA 8280 EX8/24 Part.1 R17962755, ANZ.

90 Guthrie, *op. cit.* (note 2), 43–4.

91 Biographical Notes on and Service Records of Dr. David Winn Hoodless, O.B.E, D.W. Hoodless Papers, NAF.

92 Enclosure No. 2 of Council Paper No. 11 Central Medical School, 1927. WPHC4/IV 3569/1926, WPA.

93 Legislative Council, Council Paper No. 40 Annual Medical and Health Report, Medical Department. 1938, NAF, 28.

94 Central Medical School Advisory Board: Forwards copies of minutes of various meetings. WPHC4/IV 3035/1928, WPA.

95 Central Medical School, Suva, Fiji. Annual Report for 1931. RF RG5 S3.419 B162 F1983, RAC.

96 From Hoodless to the Chairman of CMS Advisory Board, 14 October 1930, WPHC4/IV 3102/1930, WPA.

97 From V.W.T. McGusty, the Acting Chief Medical Officer to the Acting Colonial Secretary, 14 November 1934. WPHC4/IV 103/1934, WPA.

98 Minutes of Proceedings of an Extraordinary Meeting of the Central Medical School Advisory Board, 11 April 1934. WPHC4/IV 103/1934, WPA.

99 Minutes of Proceedings of a Meeting of the Central Medical School Advisory Board, 4 January 1934, WPHC4/IV 103/1934, WPA.

100 Photographs of the CMS graduation ceremony in 1939 from Margaret W. Guthrie’s private collection of David Hoodless papers in Auckland, New Zealand; Sir Harry Luke , From a South Seas Diary 1938–1942 (London: Nicholson & Watson, 1945), 135 .

101 From Harry Luke to the Secretary of State for the Colonies, 14 December 1939, CO 83/235/15, TNA.

102 Fiji Times and Herald, 24 December1940, CO 83/235/13, TNA.

103 Legislative Council, Council Paper No. 40, Annual Medical and Health Report, Medical Department. 1938, NAF, 27.

104

Maile Arvin , Possessing Polynesians: The Science of Settler Colonial Whiteness in Hawai‘i and Oceania (Durham: Duke University Press, 2019), 1–34 .

105

Bronwen Douglas , ‘Foreign bodies in Oceania’, in Bronwen Douglas and Chris Ballard (eds), Foreign Bodies: Oceania and the Science of Race 1750–1940 (Canberra: ANU Press, 2008), 3–30 .

106 Legislative Council, Council Paper No. 40, Annual Medical and Health Report, Medical Department. 1938, NAF, 27.

107 Enclosure No. 2 of Council Paper No. 11, Central Medical School, 1927. WPHC4/IV 3569/1926, WPA.

108 From Assistant Director of Education to the Secretary of WPHC, 27 September 1927, WPHC4/IV 3569/1926, WPA.

109 Correspondence on the Subjects of the Syllabus and the Four-Year Course, ACGA 8280 EX8/24 Part.1 R17962755, ANZ.

110 Central Medical School, Council Paper No. 54, Medical Department, 1934. ACGA 8280 EX8/24 Part.1 R17962755, ANZ.

111 From the Resident Commissioner to the High Commissioner, 25 April 1938, WPHC4/IV 1584/1937, WPA.

112 From Acting Secretary to the High Commission to the Central Medical Authority, Fiji, 15 March 1940, WPHC4/IV 1210/1940, WPA.

113 From Hoodless to the Secretary of WPHC, 13 March 1940, WPHC4/IV 1210/1940, WPA.

114 From Hoodless to the Secretary of WPHC, 10 November 1947, WPHC4/IV 1210/1940, WPA.

115 Minute, 10 June 1938. WPHC4/IV 1584/1937, WPA.

116 Sir A. Grantham to the Secretary of State for the Colonies, 15 December 1945, CO 83/241/6, TNA.

117 Central Medical School, Council Paper No. 54, Medical Department, 1934. ACGA 8280 EX8/24 Part.1 R17962755, ANZ.

118 Rules made by the Chief Medical Officer under section 10 of Ordinance No. 11 of 1888, Governing the conduct of Native Medical Practitioners, WPHC4/IV 1326/1919, WPA.

119 Widmer, *op. cit.* (note 2), 57–80.

120 Leckie, *op. cit.* (note 87), 70.

121 Appendix, Extract from the Annual Report for 1933–1934 of the Public Health Officer of American Samoa, ACGA 8280 EX8/24 Part.1 R17962755, ANZ.

122 Extract from page 42 of ‘The Colonial Empire in 1937–1938’ rendered by the Secretary of State to Parliament, F48 218, NAF.

123 Personal note from Lambert to Mr. Berendson, 18 January 1934, ACGA 8280 EX8/24 Part.1 R17962755, ANZ.

124 From the Secretary to the Resident Commissioner, 15 September 1937, WPHC4/IV 1584/1937, WPA.

125 From the Resident Commissioner to the High Commissioner, 25 April 1938, WPHC4/IV 1584/1937, WPA.

126 Western Pacific Health Service 3^rd^ Quarterly Report, 1930, RF RG5 S3.419 B162 F1980, RAC.

127 Extract from page 42 of ‘The Colonial Empire in 1937–1938’ rendered by the Secretary of State to Parliament, F48 218, NAF.

128 Extract from Official Report, 25 July 1938, F48 218, NAF.

129
*Native Medical Practitioner,* 3, 1 (1939), WPHC4/IV 438/1934, WPA.

130 Brewster, *op. cit.* (note 2), 408–12.

131 Fry, *op. cit.* (note 2), 184–8.

132

Ratu Sir Kamisese Mara , The Pacific Way: A Memoir (Honolulu: University of Hawai‘i Press, 1997), 18 .

